# Metabolic changes in aging humans: current evidence and therapeutic strategies

**DOI:** 10.1172/JCI158451

**Published:** 2022-08-15

**Authors:** Allyson K. Palmer, Michael D. Jensen

**Affiliations:** 1 Robert and Arlene Kogod Center on Aging and; 2 Division of Endocrinology, Diabetes, Metabolism, and Nutrition, Mayo Clinic, Rochester, Minnesota, USA.

## Abstract

Aging and metabolism are inextricably linked, and many age-related changes in body composition, including increased central adiposity and sarcopenia, have underpinnings in fundamental aging processes. These age-related changes are further exacerbated by a sedentary lifestyle and can be in part prevented by maintenance of activity with aging. Here we explore the age-related changes seen in individual metabolic tissues — adipose, muscle, and liver — as well as globally in older adults. We also discuss the available evidence for therapeutic interventions such as caloric restriction, resistance training, and senolytic and senomorphic drugs to maintain healthy metabolism with aging, focusing on data from human studies.

## Introduction: epidemiology of metabolic aging

The study of aging has long been linked with the study of metabolism, as early theories pointed to the rate of metabolism and by-products of metabolism as drivers of aging processes. The earliest recognized interventions that caused life span extension in model organisms targeted nutritional and metabolic pathways. A more nuanced view of aging mechanisms has since emerged that identifies dysregulated metabolism as one of many hallmarks of aging (1, 2).

Epidemiologic studies of the oldest old humans, centenarians, strive to identify unifying lifestyle elements, nutritional patterns, and genetic or metabolomic signatures that clearly underlie longevity. Certainly, the female sex confers a longevity advantage, as centenarians are disproportionately female (85%–90%) (3). This pattern of increased female longevity is seen across species; however, its underpinnings are incompletely understood. Social engagement, diet composition, and fasting patterns have been identified as factors that may confer longevity on a population level. Indeed, caloric restriction is known to promote longevity and delay the onset of age-related disease in multiple species (4). The people of Okinawa, who before the influence of a Western diet ate only 83% of the average calories consumed by the mainland Japanese population, were observed to have a longer life span and lower mortality from coronary artery disease and cancer than mainland Japanese or American people (5).

Long-lived humans may have some advantage in glucose handling. In one study, human centenarians (>100 years old) had better insulin sensitivity than did younger controls >75 years of age (6). Insulin sensitivity is associated with healthy aging across species, and in fact modifications in glucose signaling pathways were some of the first interventions to lead to life span extension in model systems including yeast and *Caenorhabditis* *elegans*. Similarly, inhibition of the growth hormone (GH) axis is associated with longevity in model systems. However, lifelong GH deficiency is also accompanied by smaller body size, which in humans may confer undesirable effects such as adipose tissue accumulation and intellectual deficiency (7). Body size is typically inversely correlated with longevity (such as in dogs), but this does not seem to be the case in humans.

## Anatomy and physiology of age-related changes

**Figure 1:**
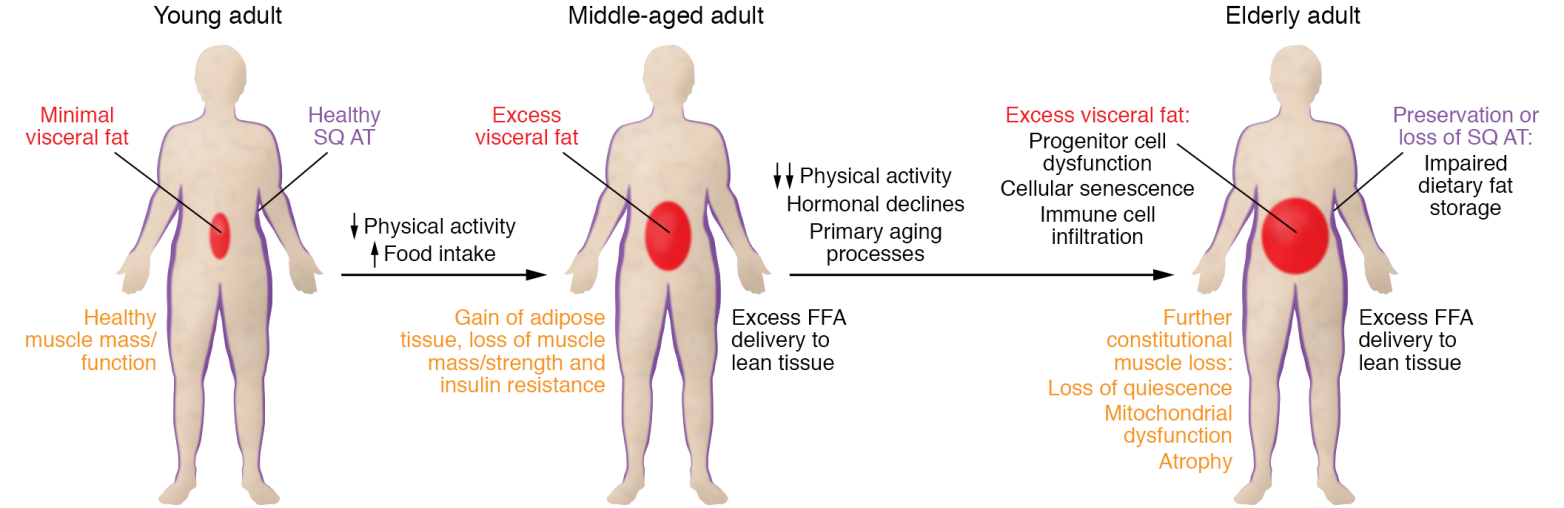
Body composition and metabolic changes with aging. The transition from healthy, active young adulthood (left), with healthy amounts and function of adipose tissue/muscle, through a sedentary lifestyle and weight gain shift to middle age (middle) to more extreme old age (right) is depicted. With a sedentary lifestyle and plentiful food, adults accumulate excess visceral fat, develop adipocyte hypertrophy in subcutaneous fat, and lose muscle mass and strength. Increased adipocyte size is associated with excess release of free fatty acids (FFAs), which have been shown to cause insulin resistance and other metabolic abnormalities; excess visceral fat causes excess FFA delivery to the liver. With extreme old age comes reduced anabolic hormones, and these reductions combined with direct effects of aging and further declines in activity result in more muscle atrophy and greater adipose tissue dysfunction. There are primary aging mechanisms at both the cellular and the organismal level. SQ AT, subcutaneous adipose tissue.

Aging in adults is associated with weight gain as indicated by both cross-sectional (reviewed in ref. 8) and longitudinal studies. Between the ages of 40 and 66, body weight in both men and women increases at an average rate of 0.3 to 0.5 kg per year and then remains stable or even continues to increase until the age of 70 (reviewed in ref. 8). However, some longitudinal studies find small declines in body weight (not in excess of 0.3% per year) in older men and women after the age of 60. Changes in body weight represent both gains in body fat and reductions in lean tissue (Figure 1). As judged by cross-sectional studies, body fat increases by an average of 1% per year in both men and women beginning as early as the fourth decade of life, although in extreme old age body fat may decline (9). The loss of lean tissue occurs in skeletal muscle and organs such as liver (10). These body composition changes result in changes in metabolic functions, because lean tissue is the primary determinant of energy requirements.

Resting energy expenditure (REE) decreases with age (11–13), and the decrease is out of proportion to the lesser amounts of lean tissue that are observed in older adults (13); we estimate that REE decreases by approximately 4 kcal/y even after adjustment for body composition (data from ref. 14). Another component of daily energy expenditure is the thermic effect of food (TEF), which is the increase in energy expenditure after meals incurred by the energy costs of digestion, absorption, and storage of nutrients. The TEF includes facultative responses that can approximately double the obligate thermogenesis related to TEF (15). The typical estimate is that TEF accounts for 10% of daily energy expenditure when humans are in energy balance (16). The TEF is reduced in older persons compared with the young by approximately 1% (12). The remaining component of daily energy expenditure — the thermic effect of physical activity — is, on average, reduced in older adults (17). This is due to lesser amounts of physical activity in older persons, not reductions in energy expenditure per unit of work (energy efficiency); older adults may actually be less energy efficient than the young during exercise (18).

## Tissue-specific changes in structure and function with aging

### Adipose tissue.

Although humans have many different adipose tissue depots, which in turn have varying metabolic functions, the three major depots are upper body subcutaneous fat, lower body subcutaneous fat, and visceral fat (19). Visceral fat is composed of the omental and mesenteric depots, whose venous blood flow is into the portal vein, thus directly impacting the liver. The two primary metabolic functions of adipose tissue with regard to fat metabolism are the storage of dietary fat following meal consumption and the release of fatty acids to meet energy needs. These depots differ with respect to these functions. For example, upper body subcutaneous adipose tissue stores dietary fat more effectively than lower body adipose tissue (20), and the storage of fat visceral adipose tissue varies widely depending on the individual’s amount of visceral fat; meal fat storage in visceral fat is more efficient in adults with small amounts of visceral fat store than in those with more visceral fat (21). There are also differences in the release of free fatty acids (FFAs) via lipolysis between upper body and lower body subcutaneous fat; lipolysis rates per kilogram fat are greater in upper body than lower body fat (22). Increasing amounts of visceral fat are related to greater hepatic FFA delivery from visceral fat (23), which can have more selective and adverse effects on liver. Thus, differences in the distribution of body fat can have major effects on the tendency toward metabolic dysfunction.

The increase in body fat with age is not uniform. Virtually every study using body composition techniques that measure fat distribution has found that older persons have more “central” (visceral and upper body subcutaneous) fat relative to total body fat. Data from cross-sectional studies suggest that body fat increases with age, up to a point at which body fat starts to decline. Older women have 300% more visceral fat than young women, but only 20% greater upper body subcutaneous fat and 45% more leg fat than young women (24). Older men have over twice as much visceral fat as young men, but only approximately 30% more upper body subcutaneous fat and no more leg fat (24). With more extremes of old age there is a progressive loss of peripheral fat (legs and arms) in some individuals (9), suggesting an inability of the body to maintain adequate subcutaneous adipose tissue mass to store lipids (8, 25).

The change in total body fat and body fat distribution is associated with changes in adipose morphology. Several investigators have noted that adipocyte size is greater in older populations (26–28). However, the adipocyte size reported in these studies appears compatible with the greater body fat content of the volunteer populations, and in a longitudinal study with an average of 13 years of follow-up, adipocyte size tended to decrease rather than increase in women first studied when they were in their 40s (29). Thus, the larger adipocytes in older adults are probably related to more total body fat and a central fat distribution. In younger populations, a central fat distribution (30) and increased fat cell size (31) are associated with abnormal regulation of adipose tissue lipolysis, which results in greater FFA release rates. This is important, because excess FFA availability can result in insulin resistance (32), greater hepatic VLDL production (33), and excess insulin secretion (34).

It is also true in older populations — greater total body and central adiposity are accompanied by greater FFA release (24, 35). In vivo studies have shown that both fasting (24) and insulin-suppressed (35) systemic lipolysis is greater in older adults than in the young. Whether this is related to more obesity/central obesity in the older adult population, as opposed to older age per se, was not reported.

In addition to tendencies toward more body fat and more central body fat with aging, women undergo menopause sometime in their 40s or 50s, and serum testosterone decreases in men as they age (36). In both sexes, serum dehydroepiandrosterone (DHEA) concentrations decrease with age, and this hormone has been proposed to mediate some of the metabolic declines with aging. The potential roles of these hormonal changes in adipose tissue function have been studied. In women, estrogen deficiency results in a 10%–20% increase in lipolysis (37), although in men there appears to be little effect of decreased testosterone on FFA release rates (38–40). Two years of DHEA replacement in older males and females with low serum DHEA concentrations had no effect on systemic FFA metabolism (40). Thus, the majority of the changes in adipose tissue lipolysis with aging appear to be more related to the greater central adiposity that is seen in older adults, rather than hormonal changes.

The other aspect of adipose tissue function that can relate to metabolic abnormalities with aging is the ability of adipose tissue to store fatty acids. First, changes in regional storage can potentially contribute to changes in body fat distribution. Second, reduced efficiency of fat storage results in greater postprandial chylomicronemia that can shunt dietary fatty acids into lean tissues, where they compete with glucose as a metabolic fuel. Meal fat storage in adipose tissue is less in older adults than in the young (24), whereas meal fat oxidation, which occurs in lean tissues, is greater in older adults (24). This is consistent with the concept that aging is accompanied by greater exposure of lean tissue to both FFA and dietary fatty acids, which may contribute to “lipotoxicity.”

Different adipose depots have different abilities to store fatty acids (41, 42), and there are sex-specific patterns of fatty acid storage (20, 43). Typically, there is a preferential storage of meal fat in upper body compared with lower body adipose tissue in young, healthy men and women (20). Older men with reduced testosterone and DHEA have a marked blunting of this pattern of meal fat storage (24), and this “youthful” distinction in regional meal fat storage was partially normalized in older men treated for 2 years with testosterone replacement therapy (24). Additional evidence for the effects of sex steroids on adipose tissue function comes from the finding that postmenopausal women have greater fatty acid storage in total and lower body subcutaneous adipose tissue than age-matched premenopausal women (44). This effect appears to be related to greater increases in abdominal adipose tissue lipoprotein lipase activity in response to meals (44) and greater abdominal adipose tissue activity of one of the key enzymes in triglyceride synthesis, diacylglycerol acyltransferase (44). Interestingly, in this study the greater adipose meal fat storage in postmenopausal females was accompanied by lesser postprandial fat oxidation than in premenopausal females (44). In men, testosterone deficiency results in greater storage of meal fat and endogenous fatty acids in lower body subcutaneous fat, which is associated with increased regional adipose tissue acyl-CoA synthetase activity (39). Thus, changes in the tendency of older adults to store dietary fat are both region- and sex-specific. Uncoupling the effects of aging, hormonal changes, and body fat gain on these functions is not easy, and only with the more severe peripheral lipoatrophy of aging is there perhaps a clearer case to be made for aging per se.

Several approaches have been used to understand the cellular nature of these changes. For example, there is some evidence that reduced physical activity contributes to adipose tissue dysfunction in older adults; physical activity training reduced some indices of adipose tissue inflammation in older women (45). However, the extent to which adipose tissue inflammation in humans contributes to adipose tissue dysfunction with regard to one of its main roles — lipolysis — has recently been questioned (31). Other approaches include the study of isolated adipocytes collected from older adults. For example, reduced ex vivo lipolysis has been found when adipocytes from older adults are studied (27, 29), although Nicklas et al. did not observe that differences in ex vivo lipolysis measures were reflected in changes in plasma FFA concentrations (27). It may be that the greater body fat content and, perhaps, in vivo adipose insulin resistance (46) explain the apparent disconnect between in vivo and ex vivo studies; studies of adipocytes collected from older adults may not reflect whole-body physiology.

**Figure 2:**
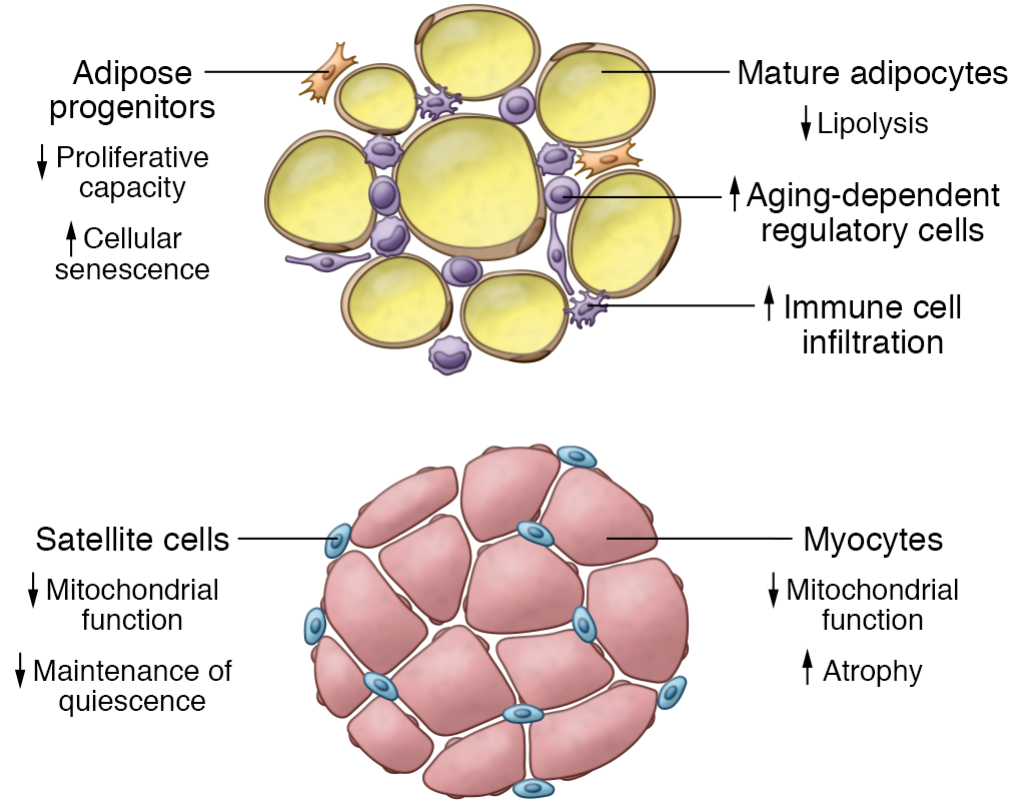
Cellular changes in adipose and skeletal muscle with aging. In adipose tissue, aged progenitor cells have reduced proliferative capacity and undergo more cellular senescence, and adipocytes’ ability to undergo lipolysis is diminished. Immune cell infiltration increases with age, and accumulation of aging-dependent regulatory cells is seen. In skeletal muscle, mitochondrial function is decreased with aging, satellite cells lose ability to maintain quiescence, and myocytes undergo atrophy.

Another helpful technique to study adipose function with aging is the study of adipose stem cells. Adipose progenitors isolated from older adults exhibit diminished proliferative and migratory capacity, reduced ability to incorporate lipids, increased oxidative stress, and features of cellular senescence (25, 47–50) (Figure 2). Cellular senescence and progenitor cell function may directly diminish the ability of subcutaneous adipose tissue to store lipids in aging (51, 52). Comparisons of subcutaneous versus visceral adipose progenitors have been done in model organisms; however, more research is needed to determine whether differences between depots in humans can explain the redistribution of adipose tissue in human aging.

Recently, a novel subpopulation of cells named aging-dependent regulatory cells (ARCs) was identified through single-cell RNA-Seq analysis of subcutaneous adipose tissue (53). ARCs are proinflammatory and inhibit differentiation and proliferation of neighboring progenitor cells, but are distinct from senescent cells in that they can proliferate. Interestingly, these cells were found only in subcutaneous, and not in visceral, adipose tissue in mice. It is not yet known whether they exist in visceral adipose tissue in humans. Nguyen et al. (53) proposed that ARCs may be involved in the loss of subcutaneous adipose tissue with age by inhibiting adipogenesis.

Adipose tissue is composed of not only adipocytes and adipocyte progenitor cells, but a complex milieu of immune cells including but not limited to NK cells, T cells, eosinophils, and macrophages. Proinflammatory immune cells accumulate in adipose tissue with aging and have been linked to development of systemic chronic low-grade inflammation. There are limited data in humans regarding the effects of aging on adipose immune cell composition and function. Eosinophil abundance in human adipose tissue appears to have a negative correlation with age, and studies in mice indicate that eosinophils may play a role in mitigating age-related adipose tissue inflammation (54). Adipose tissue macrophage abundance correlates with adiposity as well as adipocyte size and therefore may be expected to increase with aging. However, many studies describing this association were conducted in the context of obesity, and therefore the impact of aging alone is unknown (55, 56).

Adipose tissue plays a major role in endocrine signaling via secretion of adipokines. One such adipokine, adiponectin, which is associated with lower risk of metabolic syndrome in older adults (57, 58), is increased in centenarians and their children compared with non-centenarians (59). Data from animal models suggest that maintenance of adiponectin levels promotes metabolic health and prolongs life span (60). However, some studies in humans have shown a relationship between higher adiponectin levels and sarcopenia, frailty, and even mortality (61–63). There is some speculation that higher levels of adiponectin may be secondary to compensatory mechanisms in response to inflammation and oxidative stress, or may be related to adiponectin resistance, but further study is needed to clarify these points.

### Muscle.

Skeletal muscle is an important tissue for glucose and fatty acid metabolism, as well as a major site of body protein. Lean tissue, typically constituting 53% and 47% of muscle in men and women, respectively (64, 65), is reduced in older adults, especially in older compared with young women (24). This difference is even more evident when imaging techniques such as CT or MRI are used. Cross-sectional comparisons between young and older study participants suggest that by age 60 humans will lose approximately 0.7% to 0.8% of muscle per year. At age 60, men have 14% less leg muscle than 20-year-old men (66), and older adults will lose approximately 0.8% of trunk muscle mass per year (67). These changes have been confirmed in longitudinal studies; leg muscle mass declined by 1% per year in older women and men (68), and trunk muscle decreased by 0.2% to 0.4% per year in older women and 0.4% to 0.8% per year in older men (69). Thus, muscle loss includes both locomotive and postural muscles. Together with loss of muscle mass, there is often an increase in intramuscular adipose tissue (68) — so-called marbling. This is to be distinguished from intramyocellular lipids, which have also been reported to be increased with aging (10, 70, 71). Intramuscular adipose tissue is a marker of, but unlikely to be a major cause of, muscle dysfunction. This is because intramuscular adipocytes, when present, are adjacent to blood vessels outside the perimysium; that is, there are no adipocytes inside muscle fascicles (72). Because there is no portal system in skeletal muscle, intramuscular adipocytes are unable to provide fatty acids or lipokines to myocytes directly.

The lesser amount of skeletal muscle in older adults is accompanied by reduced muscle function. There are progressive declines in peak VO_2_ (ref. 73; and reviewed by Russ and Lanza, ref. 74) and strength (8, 70, 73, 75) with age. The loss of strength (2.5%–4% per year) is substantially greater than the loss of muscle (1% per year), implying that reduced muscle function plays an important role in the loss of strength (75) with age. An autopsy study indicated that muscle atrophy begins around 25 years of age and thereafter accelerates owing to a loss of fibers more than to a reduction in fiber size (76), although others have suggested that reduced fiber size is a more important determinant of muscle atrophy (66). The loss of muscle has implications for both mobility and metabolic regulation (muscle is the primary site of insulin-mediated glucose storage and oxidation), making the physiological explanation for age-related muscle loss an important area of study.

Muscle proteins are constantly turning over; older, damaged proteins are replaced by newly synthesized proteins. Normally, muscle protein synthesis (anabolism) is stimulated by meal consumption and resistance exercise (77). The amino acids, especially leucine, provided by meal consumption are thought to be the main drivers of this anabolic response. The combination of resistance exercise and meal protein consumption is synergistic with respect to muscle protein synthesis (77). Insulin acts to restrain muscle protein breakdown, even in the fasting state (78). Aging has been shown to be associated with resistance to the anabolic effects of both resistance exercise and meal consumption, as well as resistance to the anticatabolic effects of insulin (77).

A substantial portion of the loss of muscle mass and strength with aging is due to reduced physical activity. Adults who maintain high levels of physical activity into their 60s and above have less body fat and more muscle, are more fit, and are stronger (28, 70, 79). That said, even those who maintain such high levels of physical activity suffer declines in fitness and strength over time, indicating a primary aging effect. Do these primary effects of aging account for the observed insulin resistance with respect to glucose metabolism that has been described in older adults (80)?

Although insulin resistance with respect to glucose metabolism was once thought to be primarily an effect of aging, it is now known that the greater amounts of body fat (81) and particularly visceral fat (82) are much better predictors of insulin resistance than is age. In fact, after accounting for body fat and fat distribution, age and fitness do not predict insulin action with respect to glucose metabolism (82). There may also be abnormalities of muscle lipid metabolism associated uniquely with aging, although not as many studies have been done. In response to leg exercise, leg muscle of older men utilizes more plasma FFA and less intramuscular triglycerides than that of young men (71). The intramyocellular content of ceramides and diacylglycerols, especially of the saturated fatty acid varieties, is greater in older than in younger men (83). Plasma FFA concentrations correlate with intramyocellular lipid content in some thigh muscles; intramyocellular lipid content inversely correlated with measures of strength in young, but not in older, adults (84). Although there are some differences between young and older men in several proteins/enzymes that regulate fatty acid metabolism (83), it is unknown whether these are due to differences in age, adiposity, or fitness. In summary, much of the insulin resistance with respect to glucose metabolism that was attributed to aging is, in fact, more strongly related to the tendency for older adults to develop central obesity. Maintaining a healthy amount of body fat with aging reduces the risk of central obesity–related metabolic disorders, and maintaining high levels of physical activity (including resistance exercise) can partially offset the progressive loss of muscle mass.

Activation of muscle progenitor cells, or satellite cells, is necessary for muscle regeneration, given that myofibers are terminally differentiated. Satellite cells exist in a quiescent state between the sarcolemma and basal lamina in muscle. When activated in response to damage or stress, satellite cells proliferate and eventually fuse, forming new myofibers. A subset of cells reenter the quiescent state, thereby maintaining the progenitor cell pool. In vitro analysis of satellite cells obtained from human muscle indicates that with aging of the individual, satellite cells maintain their capacity to proliferate and differentiate (85). However, immunohistochemical analysis of muscle biopsies indicates that the number of satellite cells per muscle fiber decreases with age (86). Aged human satellite cells also have reduced ability to remain quiescent and exhibit features of cellular senescence including p16^INK4a^ expression (87), which may contribute to depletion of the progenitor cell pool over the life span. In addition to cell-intrinsic factors, age-related changes in the progenitor cell niche, including the extracellular matrix, may also affect the ability to maintain quiescence (88, 89) (Figure 2).

Mitochondrial function, including ATP synthesis and oxidative capacity, decline with aging in skeletal muscle (90). It is not entirely clear whether these changes are related to primary aging or are secondary to decreased physical activity (91, 92); however, recent evidence suggests that both factors contribute (18), likely to varying degrees between individuals owing to heterogeneity along the life span. Mitochondrial DNA (mtDNA) accumulates mutations with aging, and mtDNA copy numbers, which are associated with oxidative capacity, do seem to decrease with aging independent of changes in activity (93). These changes may lead to reduced synthesis of mitochondrial proteins, in turn impacting metabolic capacity.

A number of pathways have been implicated in the muscle atrophy of aging using rodent models. These include activation of activating transcription factor 4 (ATF4), which, through a complex pathway (94), appears necessary for muscle atrophy to occur with aging (mice that lack ATF4 in skeletal muscle fibers maintain muscle strength and mass into old age; ref. 95); and 15-hydroxyprostaglandin dehydrogenase, the prostaglandin E_2_–degrading enzyme, which, when inhibited, increased aged muscle mass, strength, and exercise performance in mice (96).

### Liver.

Although how the liver changes with age in humans has not been studied as much as how skeletal muscle and adipose tissue change, the central importance of the liver in metabolic function is such that what is known must be considered. Liver mass and liver blood volume have been found to be less in older adults than young; a 20% to 40% decrease in both has been reported in older adults (reviewed by Kim et al., ref. 97), although most standard biochemical tests used to reflect liver function remain normal. It has also been reported that older adults are more likely to have increased liver fat and that greater amounts of liver fat are associated with metabolic abnormalities (10). However, the greater amounts of liver fat in older adults may well be explained by their greater tendency to have increased total body and visceral fat.

Most of the differences between younger and older adults with respect to hepatic glucose metabolism can be accounted for by differences in body fat and body fat distribution (82), although increased hepatic insulin clearance combined with decreased peripheral insulin clearance appears to be linked with aging more than body composition or fitness (82, 98). Thus, the evidence suggests that the role of the liver in modulating how insulin secretion results in peripheral insulin delivery is directly modulated by aging.

Some of the other factors that may contribute to abnormalities in hepatic metabolism with aging are hormonal changes. In addition to the changes in sex steroids outlined above, aging is associated with delayed and impaired insulin secretion (35, 98), greater fasting plasma cholecystokinin (CCK) and glucagon-like peptide-1 (GLP-1) concentrations, and greater CCK and GLP-1 responses to protein ingestion (99). Thus, untangling the roles of hormonal changes that accompany aging from age-specific differences in liver function in humans is not easy.

## Therapeutic interventions

Therapeutic interventions under investigation to improve metabolic function with age in humans include a range of dietary modifications/fasting patterns, aerobic and resistance training, and a limited number of pharmacologic strategies. Caloric restriction (CR), the reduction of calorie intake without malnutrition, has been shown to increase life span and improve metabolic health in multiple model systems. A study of CR in humans (the Comprehensive Assessment of Long-term Effects of Reducing Intake of Energy [CALERIE]) showed weight loss, reduction in total daily energy expenditure, and reduction of the inflammatory markers TNF-α and CRP (100). A follow-up study showed reduction of subcutaneous and visceral abdominal adipose tissue mass as well as less intramyocellular lipid content after calorie restriction (101). Insulin sensitivity was improved initially but was not different in the CR group after 2 years, the significance of which is unclear given that the study was conducted in healthy normal to moderately overweight individuals (BMI between 22 and 28 kg/m^2^) (100). Given the beneficial effects of CR, but the considerable effort required to achieve this intervention in the short and especially the long term, much investigation has been conducted into pharmacologic interventions to mimic CR (reviewed by Madeo et al., ref. 102). One of these interventions has been resveratrol. Randomized trials of resveratrol have documented changes in muscle mitochondrial function and adipose gene expression (103, 104) but no clinically meaningful responses.

As noted above, older adults who exercise regularly have less central body fat, better muscle function, and better insulin sensitivity than sedentary older adults (45, 66, 70, 79), and exercise training, with or without weight loss, improves muscle mass and function in older adults (26, 68). Satellite cell content and activation increase in response to exercise (105–107), and data from animal studies are beginning to elucidate the mechanisms by which exercise may improve progenitor cell function (108). Metabolic abnormalities with aging seem to be most readily addressed by aggressive resistance training, which can offset some, but not all, of the anabolic resistance in muscle (77). Thus, of the interventions that have been studied, exercise training appears to have the most wide-ranging benefits when it comes to muscle and adipose tissue health, the latter at least partly through prevention of excess fat gain.

The beneficial effects of resistance exercise on skeletal muscle mass and strength are enhanced by provision of adequate protein intake (109). Increased protein intake is needed for older adults to achieve a response to exercise training similar to that of their younger counterparts, owing to anabolic resistance (110). Leucine supplementation and β-hydroxy-β-methylbutyrate (HMB) supplementation may be beneficial for increasing muscle mass in older adults with sarcopenia independent of exercise (109). Other nutritional approaches to enhance muscle mass and function with aging include supplementation with omega-3 fatty acids (111, 112), although it is unclear through what mechanism this benefit occurs.

Several pharmacologic approaches to improve muscle function with aging have been tried; the outcomes of the resveratrol trials were mentioned above. Pioglitazone, given in combination with a weight loss program with or without resistance exercise, results in greater loss of visceral fat in older men (113), and perhaps greater strength gains when combined with resistant training in older women (114). The pathways that account for this effect may include normalization of PI3K/Akt signaling and increased adiponectin expression, leading to reduction of muscle protein degradation and autophagy and improved progenitor cell function (115–117). Many preclinical investigations are ongoing to identify mechanisms of age-related muscle atrophy and dysfunction and to test novel therapeutics targeting newly discovered pathways. For example, therapeutics that target the atrophy mechanisms downstream from ATF4 have included ursolic acid and tomatidine, which promote skeletal muscle hypertrophy (95) and appear to increase mTORC1 activity in skeletal muscle. However, translation to successful clinical applications has so far been limited (118).

Senescent cells have emerged as an attractive therapeutic target in the prevention and treatment of age-related and metabolic disease (119). A recent study in humans with diabetic kidney disease demonstrated the ability of senescent cell–targeting, or senolytic, drugs to remove senescent cells from human adipose tissue (120). Other strategies under development to target the effects of senescent cells include “senomorphic” drugs, which aim to reduce the senescence-associated secretory phenotype, and emerging antibody-mediated therapies (121). Further study is needed to determine whether senescence-targeting therapies can improve body composition and metabolic health in aging humans.

## Conclusion

Aging is associated with increased adiposity, and the prevalence of obesity is increasing in the older adult population. Older adults who maintain an active lifestyle and avoid gaining excess fat are largely spared from the typical metabolic syndrome/insulin resistance features that have been attributed to aging. However, the tendency toward loss of peripheral adipose tissue and the muscle atrophy that accompanies aging cannot be completely prevented. Adequate dietary protein, perhaps omega-3 fatty acids, and exercise training are the current mainstays of preventing metabolic abnormalities with aging. Correcting pathological hormonal deficiencies can also result in positive metabolic changes. Therapeutics that mitigate the effects of senescent cell accumulation or target stem cell populations in adipose tissue and atrophy pathways in muscle are on the horizon. These could combine to further improve the metabolic health of older adults.

## Version 1 08/15/2022

Electronic publication
